# TRIzol treatment of secretory phase endometrium allows combined proteomic and mRNA microarray analysis of the same sample in women with and without endometriosis

**DOI:** 10.1186/1477-7827-9-44

**Published:** 2011-04-06

**Authors:** Amelie Fassbender, Peter Simsa, Cleophas M Kyama, Etienne Waelkens, Attila Mihalyi, Christel Meuleman, Olivier Gevaert, Raf Van de Plas, Bart de Moor, Thomas M D'Hooghe

**Affiliations:** 1 Leuven University Fertility Centre, Department of Obstetrics & Gynaecology, University Hospital Gasthuisberg, Leuven, Belgium; 2 Division of Reproductive Health and Biology, Institute of Primate Research, P.O. Box 24481-00502 Karen, Nairobi, Kenya; 3 Biochemistry Section, Department of Molecular Cell Biology, Campus Gasthuisberg, Leuven, Belgium; 4 Department of Electrical Engineering, ESAT-SCD, K.U.Leuven, Kasteelpark-Arenberg 10, B-3001 Heverlee, Belgium

## 

Since publication of our article [1], we have realised that we did not include the full data in Table 1 and missed the word (TOF) in the result/discussion section. We have provided here the adapted sentence and another version of the table, including all the information intended.

**Table 1:** The representative molecular weights of the proteins identified in the mRNA Microarray study [Thirteen] [2]

Protein	Mass in Da
**Osteoglycin (OGN/4969)**	33,922
**Interleukin-6 signal transducer (IL6ST/3572)**	isoform 1 103,537 isoform 2 37,499
**Cytochrome P450, Family 2, Subfamily J, polypeptide 2 (CYP2J2/1573)**	57,611
**Carboxypeptidase E (CPE/1363)**	53,151
**Fibronectin 1 (FN1/2335)**	**different isoforms**
	1. 262,607
	2. 71,943
	3. 259,198
	4. 222,944
	5. 243,316
	6. 240,477
	7. 268,894
	8. 252,793
	9. 246,670
	10. 239,608
	11. 262,388
	12. 221,274
	13. 249,304
	14. 249,384
	15. 272,302
**Synuclein, gamma (SNCG/6623)**	13,331
**BAI1-associated protein 2 (BAIAP2/10458)**	**different isoforms**
	1. 60,868
	2. 59,014
	3. 56,626
	4. 57,359
	5. 57,445
	6. 57,430
**Protocadherin 17 (PCDH17/27253)**	**different isoforms**
	1. 126,229
	2. 96,570
**Protein tyrosine phosphatase, receptor type, R (PTPRR/5801)**	Alpha 73,834 Da Gamma 46,581Da Delta 51,046Da

## Results/Discussion

"Therefore, we plan to repeat this study in a larger sample size including well defined endometrial samples obtained during menstrual, follicular and secretory phase, to validate the reproducibility of SELDI-TOF MS technology in these samples and to identify the protein peaks observed after proteomic analysis, which are expensive and labour intense requiring High-performance liquid chromatography or high-pressure liquid chromatography (HPLC) and matrix assisted laser desorption ionization Time-of-Flight-Mass Spectrometry (MALDI-TOF/TOF MS)."

## References

[B1] Fassbender Amelie Simsa Peter Kyama Cleophas M Waelkens Etienne Mihalyi Attila Meuleman Christel Gevaert Olivier Van de Plas Raf de Moor Bart D'Hooghe Thomas M TRIzol treatment of secretory phase endometrium allows combined proteomic and mRNA microarray analysis of the same sample in women with and without endometriosis Reproductive Biology and Endocrinology 2010 8 123 10.1186/1477-7827-8-123 20964823 PMC2987945

[B2] Sherwin JR Sharkey AM Mihalyi A Simsa P Catalano RD D'Hooghe TM Global gene analysis of late secretory phase, eutopic endometrium does not provide the basis for a minimally invasive test of endometriosis Hum Reprod 2008 23 5 1063 1068 10.1093/humrep/den078 18353903

